# Childhood Obesity and its Influence on Sleep Disorders: Kids-Play Study

**DOI:** 10.3390/ijerph17217948

**Published:** 2020-10-29

**Authors:** Antonio Manuel Sánchez-López, Jessica Pamela Noack-Segovia, Ana María Núñez-Negrillo, Julio Latorre-García, María José Aguilar-Cordero

**Affiliations:** 1 Department of Human Motricity and Sports Performance, Faculty of Education, University of Sevilla, 41013 Sevilla, Spain; 2 Research Group CTS 367, Andalusia Research Plan, Junta de Andalucía, 11620 Junta De Los Rios, Spain; noackjessica1966@gmail.com (J.P.N.-S.); amnunez@ugr.es (A.M.N.-N.); juliolatorrefisio@gmail.com (J.L.-G.); mariajaguilar@telefonica.net (M.J.A.-C.); 3 Nursing Department, Universidad de Santo Tomás, 8370003 Talca, Chile; 4 Nursing Department, School of Health Sciences, University of Granada, 18071 Granada, Spain

**Keywords:** sleep apnoea, childhood obesity, respiratory polygraphy, apnoea–hypopnea index

## Abstract

*Background:* Sleep disorders are associated with overweight and obese children, and could decrease life quality with limitations to normal daily activities. The purpose of the study is to describe the prevalence of sleep disorders in a cohort of overweight/obese children using respiratory polygraphy. *Methods:* A descriptive cross-sectional study was conducted in Granada (Spain) on a sample of 98 children with overweight or obesity. The presence of sleep disorders was determined by respiratory polygraphy. *Results:* Regarding apnoea–hypopnea-index (AHI) results, 44% of affected children had severe sleep apnoea–hypopnea syndrome (SAHS), and the remaining 56% had a mild form of the disorder. With respect to oxygen-desaturation index, 56% of the same group had severe SAHS, 32% had mild SAHS, and the remaining 12% did not suffer from SAHS. Among participants, average scores of 13.8 obstructive apnoea, 7.7 central apnoea, and 13.6 hypopnoea were recorded. *Conclusions:* Respiratory polygraphy can provide conclusive results in the diagnosis of SAHS in overweight/obese children. Interventional programmes designed and implemented to reduce overweight and obesity can improve quality of sleep and life in children.

## 1. Introduction

According to the World Health Organisation (WHO), overweight and obesity in childhood are a global public-health problem, and interventions at an early age can effectively prevent it, thus reducing their incidence among adults. UNICEF, the WHO, and the World Bank reported that the global number of overweight children increased from 32 to 42 million in 2000–2013. If this trend continues, overweight prevalence among children under 5 years old will rise to 11% by 2025, with 70 million infants and young children being affected. It is important to determine the factors that predispose to the development of overweight and obesity in childhood, so that timely preventive measures can be taken. Overweight and obesity in children were linked to sleep disorders, although few well-designed scientific studies addressed this issue [1,2,3,4].

At least four clinical childhood- and juvenile-obesity phenotypes associated with sleep apnoea–hypopnea syndrome (SAHS) have been described. The risk of suffering from SAHS among overweight or obese children is more than four times higher than the risk of healthy, normal-weight children [5].

Sleep is defined as the normal suspension of consciousness in terms of electrophysiology by specific brain-wave criteria. Over 30% of a child’s life is spent sleeping, this state being critical for growth and development. For this reason, sleep pathologies and their clinical repercussions have been priority areas of scientific research in recent years [6].

The international classification of sleep disorders (ICSD-2) includes central apnoea syndrome, obstructive sleep apnoea syndrome, hypoventilation syndrome, and sleep hypoxaemia. However, other, unspecified related disorders also exist. In overweight or obese children, the most common of the aforementioned alterations is sleep apnoea–hypopnea syndrome (SAHS) [7,8,9].

Sleep disorders are a major problem in developed countries, affecting 4% of males and 2% of females of the adult population [10]. Estimated prevalence of SAHS in children is 2–6% amongst general population. However, in obese adolescents, prevalence ranges from 13% to 66% according to different studies [11]. The incidence of snoring ranges from 7% to 16.7% of all infants and children aged between 6 months and 13 years old [12,13,14].

SAHS is triggered by the partial collapse of the airway or decrease in its diameter during inhalation [11]. This pathology in children is a public-health issue of remarkable importance due to its high prevalence, its association with various chronic processes, and its capability to worsen the course of the pathologies with which it is associated [15,16].

The aim of this study is to describe the prevalence of sleep disorders in a cohort of overweight/obese children and adolescents using respiratory polygraphy.

## 2. Materials and Methods

### 2.1. Design and Sample Selection

A descriptive cross-sectional study was carried out in Granada (Spain) from 2016 to 2017 within the Kids-Play study, registered at www.clinicaltrials.gov (identifier NCT02779647) and published [17]. Participants were recruited at the paediatric clinics of 12 health centres and at the endocrinology clinics of the University Hospital Complex of Granada. Study-participation requirement was the provision of informed consent by each child’s parents or legal guardian.

The study universe was composed of 521 children with overweight or obesity who attended the paediatric and endocrinology clinics. For a 95% confidence level of *p* = 0.05 and a maximal estimation error of 10%, a sample size of n = 98 was required for each group. Overweight and obese status is defined as a body-mass index (BMI = weight/height^2^ (g/m^2^)) greater than the cut-off values established by the International Obesity Task Force for age and sex in children and adolescents [18]. In addition, body composition was measured using bioelectrical impedance. Inclusion criteria: obese children that wanted to voluntarily participate in the research and signed the informed consent. Exclusion criteria: refused to participate, hormonal problems, age <8 or >12 years, orthopaedic problems, and other problems.

### 2.2. Body Composition

The body composition of the children was measured using an InBody^TM^ 720 analyser [19]. This device obtains bioelectrical impedance to determine body composition, weight, and height. The human body is composed of water, protein, body fat, and minerals, and these components can be quantified by bioelectrical impedance. Measurement of these variables was of fundamental importance in determining the degree of overweight or obesity in the children included in the study.

### 2.3. Paediatric Sleep Questionnaire: Short-Form Spanish Language Version

The Paediatric Sleep Questionnaire was developed and validated by Ronald et al. [20], and translated into Spanish by Vila et al. [21]. It consists of two versions, a short and a more detailed form. This study uses the abbreviated version, which aims to detect sleep-related disorders. It can be used for children aged 2–18 years, and provides a very complete description of any sleep disturbances.

### 2.4. Respiratory Polygraphy

Respiratory polygraphy (RP) consists of recording and subsequent analysing respiratory and cardiac variables during sleep. It is commonly used as a means of diagnosing SAHS.

The main advantage of RP is that it is a much simpler method than polysomnography (PSG) is, but its main disadvantage is that it does not assess neurophysiological variables: electroencephalography (EEG), electrooculography (EOG) and electromyography (EMG). However, the quantity of sleep can be recorded via channels reflecting thoracic and abdominal excursion, body position, actimetry, and snoring. PSG and RP are complementary techniques that can be performed in a fully equipped sleep unit. Each technique is used to assess paediatric SAHS, but RP is more commonly used in children since it is less invasive and can be applied at home [22]. Nevertheless, in the present study, all polygraphs were obtained at the hospital in order to ensure the reliability of the obtained results.

RP equipment was the Embletta Portable Diagnostic System^TM^ [23]. Data were reviewed and analysed using Somnologica for Embletta software.

Once the patient was ready to start sleeping, sensors were placed to facilitate the recording of parameters to be analysed. The following parameters were studied:

- Flow signal, recorded using a nasal pressure transducer.
- Respiratory effort, assessed from thoracoabdominal movements and detected by two plethysmographic bands. A third band, fastening the junction box, contained the body-position sensor.
- Arterial oxygen saturation and pulse, recorded by a pulse oximeter placed on a finger.

SAHS consists of repeated occurrence of episodes of complete (apnoea) or partial (hypopnea) pharyngeal obstruction in sleep, caused by respiratory collapse. For adults, apnoea means the cessation of airflow for at least ten seconds. In children, it is defined as the duration of the event equivalent to two respiratory cycles, and is associated with a ≥90% reduction in the amplitude of the thermistor signal for over 90% of the total event. Hypopnea is said to occur when at least one of the following criteria is met [9]:

- severely reduced airflow (>50%);
- moderately reduced airflow (<50%) with >3% oxygen desaturation;
- moderately reduced airflow (<50%) with associated electroencephalographic evidence on awakening.

### 2.5. Ethical Questions

This research project was approved by the Research Ethics Committee of the province of Granada, Spain (CEI Granada).

The well-being and privacy of patients involved in the research must be among a researcher’s main concerns. It is explicitly stated that this study complies with the ethical standards issued by the Research and Clinical Trials Committee, as published in the 1964 Declaration of Helsinki (revised in Fortaleza, Brazil, 2013).

### 2.6. Statistical Analysis

Descriptive analysis of the main study variables was performed. Quantitative data are presented as mean, standard deviation, median, and percentiles. Qualitative variables are shown as percentages. All data were analysed using SPSS v.19 statistical software (IBM, New York, NY, USA).

## 3. Results

**Figure 1:**
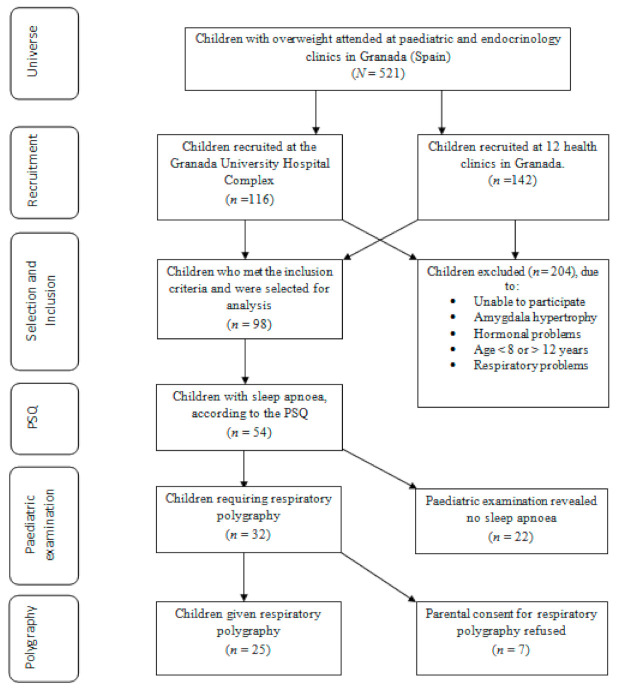
Sample selection.

Sample-selection results are outlined in Figure 1. The resulting population of 98 overweight or obesity children were asked to complete the Paediatric Sleep Questionnaire (PSQ), which revealed that 44 (44.9%) of the children did not present SAHS symptoms. The remaining 54 children presented signs of the disorder and attended the paediatric clinic where 22 children were discarded as cases after diagnosis. Respiratory polygraphy was prescribed for the remaining 32 children (31.4%). In seven cases, the procedure was not implemented because parental consent was refused. Lastly, 25 children were given respiratory polygraphy to evaluate the presence of SAHS.

Table 1 describes the characteristics of the study population [17]. The initial sample included 52 boys and 46 girls, with a mean age of 10.65 ± 1.38 years, a mean weight of 66.05 kg, and a mean height of 150.75 cm, which is equivalent to a mean BMI of 28.60 and 40.2% body fat. Of the sample, 15.6%, were overweight and 84.4% were obese. Of the children, 50% gave positive answers to more than seven questions on the short-form PSQ. This information was interpreted as evidence of possible SAHS. Therefore, these children were referred to the paediatric unit for physical examination. As a result, 32 children (29.5%) underwent a respiratory-polygraphy test in order to determine the presence or absence of SAHS. Lastly, the RP test was only administered to 25 children due to parental consent refusal in the seven remaining cases. Characteristics of the 25 children that underwent RP are presented in Table 1.

Table 2 describes the outcomes of the RP test performed on these 25 children. The following variables were studied: percentage of oxygen saturation (% SO_2_), mean heart rate (HR_mean_), maximal heart rate (HR_max_), minimal heart rate (HR_min_), apnoea–hypopnea index (AHI), total desaturation (Des_total_), and index of oxygen desaturation (Des_index_).

The presence of SAHS in children, examined at the Sleep Unit of the University Hospital of Granada, was diagnosed using the apnoea–hypopnea (AHI) and oxygen-desaturation indices. For both indices, it was assumed that a <1 value showed the absence of SAHS, a value of 1–3 corresponded to a mild form of the disorder, and a >3 value reflected a severe form of the condition. The use of these parameters is supported by previous research [24,25,26].

Table 3 shows that, according to the AHI, 44% of the tested children had severe SAHS and 56% had mild SAHS. According to the desaturation index, 56% of these children had severe SAHS, 32% had mild SAHS, and 12% did not have SAHS.

**Figure 2:**
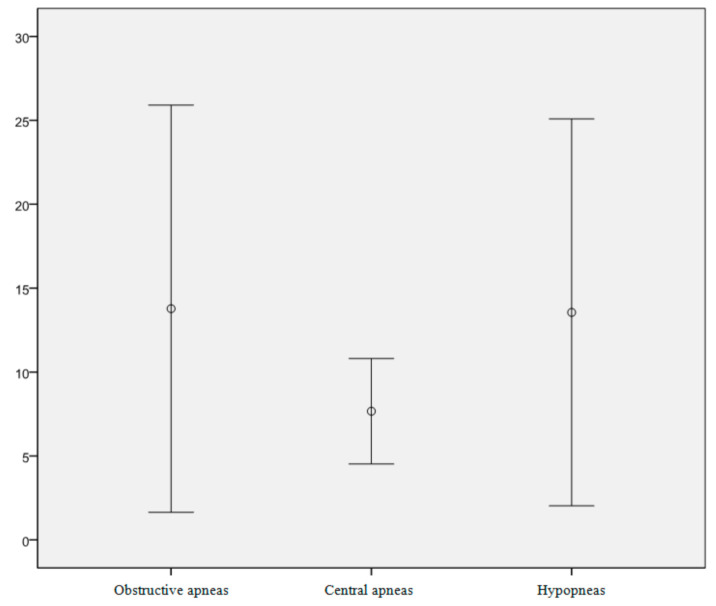
Mean numbers of obstructive apnoea, central apnoea, and hypopnea recorded in the studied children.

Figure 2 shows the number of apnoea cases recorded by RP in the study population. On average, these children presented 13.78 instances of obstructive apnoea, 7.67 of central apnoea, and 13.56 of hypopnea. Overall, disorder incidence among the participants was high.

## 4. Discussion

Few studies were conducted to analyse SAHS in overweight or obese children; in general, little is known about sleep disorders in children. Therefore, more research is needed to highlight the importance of the proper diagnosis and treatment of SAHS in this population.

SAHS was diagnosed in 25.5% of the 98 obese children included in the present study according to respiratory polygraphy, although a further 7.1% (whose parents refused consent for the RP test) presented clear signs of SAHS according to the PSQ and paediatric examination. Our study thus suggests that 32.6% of children with overweight or obesity could present SAHS. As 2–6% of healthy normal-weight children are estimated to have SAHS, we conclude that the presence of overweight increases the risk of children being affected by this sleep disorder. In this respect, Caminiti et al. found that 55% of children with obesity had SAHS, confirmed by polysomnography [11]. These values are somewhat higher than those found in our study, but they corroborate the view that obesity is a factor that is clearly associated with SAHS.

The obtained results by the RP test in the present study are similar to those reported by Wing et al. [27], who analysed the presence of SAHS in a population of 46 children with obesity (average age: 10.8 years), compared to a control population of 44 normal-weight children. The AHI scores obtained reflected an average value of 3.9 for the obese children, which is close to the 4.08 recorded in our own study. On the other hand, the oxygen desaturation index value differed considerably; 9.77 for Wing et al. vs. 2.83 in our study. Nevertheless, in general, the results are similar in both studies, in which around 30% of the children with obesity were found to have SAHS.

This pattern is corroborated by Xu et al. [28], who, in 2008, performed a sleep study on 99 obese and 99 normal-weight children, and observed a higher AHI score in the obese children. However, in their study, the cut-off value to diagnose SAHS was AHI ≥ 5, while the corresponding value was AHI ≥ 3 in our study. Another study, by Kang et al. [29], carried out in 2013, concluded that obesity is a risk factor for SAHS in children according to a polysomnography study of 495 children with symptoms of sleep disturbance, which showed that children with obesity had higher AHI scores than those of children with normal weight.

In 2014, Alonso-Álvarez et al. [30] performed a very similar study to our own using PSQ and polysomnography to study the sleep patterns of 248 obese children (average age: 10.8 years). These authors obtained an AHI score of 5.58, which is a little higher than the 4.08 we recorded. Both studies, however, clearly show that obesity is a risk factor for SAHS in children.

Opinions differ regarding the most appropriate index to be used for determining a diagnosis of SAHS. We chose to use the AHI as the main measure with the oxygen-desaturation index as a support measure, thus strengthening diagnosis. The reliability of the AHI was confirmed in previous studies, such as Masumoto et al. [31], who reported that children with AHI > 3 experienced greater sleep disturbance than those with lower AHI values. Accordingly, AHI ≥ 3 was established as a valid measure for diagnosing sleep patterns, and for differentiating between mild and severe disorders.

Lastly, respiratory polygraphy is a reliable method for the diagnosis of SAHS in children with obesity, as reported in previous studies [22,32]. One conducted by Zafanello et al. [33] in 2016 used respiratory polygraphy to examine 36 children with obesity, and found that this condition aggravated snoring, a respiratory problem that affects sleep and is related to SAHS.

The main limitation of the study was the rejection of parents to perform hospital respiratory polygraphy even though it was recommended by a sleep specialist. The main strength was the reliability of hospital respiratory polygraphy when obtaining the valid results.

## 5. Conclusions

The present study shows that childhood overweight and obesity are related to sleep disorders in general and to SAHS in particular, which were recorded using respiratory polygraphy. These disorders were manifested as snoring, nightmares, night terrors, and enuresis. On the other hand, SAHS in childhood may also be due to amygdala hypertrophy; so, before RP is performed, an examination in this respect should be conducted by the paediatrician. In the obese and overweight children in our study, the possible involvement of amygdala hypertrophy was considered and rejected. In all cases, the observed sleep disorders arose from the presence of overweight or obesity.

Another relevant finding is that these children’s parents do not generally consider SAHS to be a significant problem; this belief is especially strong among the parents of overweight or obese children. In fact, the consequences of this sleep disorder are often unknown to the families concerned. This circumstance was made evident in our study, where a high percentage of parents refused consent for a respiratory-polygraphy test to be performed on their children, despite the positive PSQ findings, confirmed by the paediatrician’s diagnosis.

According to the current and previous research, SAHS is present in 2%–6% of normal-weight children and in approximately 30% of those with overweight or obesity. However, the actual values could be even higher since many children may present this disorder but fail to be diagnosed.

Physical activity to reduce overweight and obesity levels is considered to be of critical importance in reducing SAHS incidence. For this reason, we recommend an educational intervention based on physical activity, and on providing a nutrition report to children and their families.

## Figures and Tables

**Table 1:** Descriptive sample characteristics (*n* = 25) based on bioimpedance test results.

	Weight	Height	BMI	Body Fat %
Mean (SD)	66.05 (17.39)	150.75 (11.08)	28.60 (4.05)	40.19 (5.74)
Minimum	38.80	129.00	21.43	25.10
Maximum	106.20	180.00	38.50	50.70

**Table 2:** Respiratory polygraphy results (*n* = 25). Note: % SO_2_, oxygen-saturation percentage; HR_mean_, mean heart rate; HR_max_, maximal heart rate; HR_min_, minimal heart rate; AHI, apnoea–hypopnea index; Des_total_, total desaturation; Des_index_, oxygen-desaturation index.

	% SO_2_	HR_mean_	HR_max_	HR_min_	AHI	Des_total_	Des_Index_
Mean (SD)	95.74 (1.02)	73.57 (1.79)	99.61 (10.42)	60.68 (4.70)	4.08 (3.93)	21.33 (14.79)	2.83 (1.87)
Minimum	94.40	70.60	86.50	52.00	1.00	0.00	0.00
Maximum	97.10	76.60	124.00	66.80	10.80	51.00	5.40

**Table 3:** Percentages of children with sleep apnoea–hypopnea syndrome (SAHS) according to AHI and desaturation index.

	Desaturation Index % (*n*)	AHI % (*n*)
Healthy	12 (3)	0 (0)
Mild SAHS	32 (8)	56 (14)
Severe SAHS	56 (14)	44 (11)
